# FLEXIBLE INTRAMEDULLARY NAILS IN PEDIATRIC SUBTROCHANTERIC FEMUR FRACTURE: BIOMECHANICAL STUDY

**DOI:** 10.1590/1413-785220233102e260008

**Published:** 2023-06-09

**Authors:** MÁRIO AUGUSTO FERREIRA CRUZ, LEONARDO RIGOBELLO BATTAGLION, JOSÉ BATISTA VOLPON

**Affiliations:** 1. Universidade de São Paulo, Escola de Medicina de Ribeirão Preto, Ribeirão Preto, SP, Brazil; 2. Universidade Tiradentes, Aracaju, SE, Brazil.; 3. Universidade de São Paulo, Escola de Medicina de Ribeirão Preto, Bioengineering Laboratory, Ribeirão Preto, SP, Brazil.; 4. Universidade de São Paulo, Escola de Medicina de Ribeirão Preto, Department of Orthopedics and Anesthesiology, Ribeirão Preto, SP, Brazil.

**Keywords:** Femoral fractures, Fracture fixation, intramedullary, Hip fractures, Fraturas do fêmur, Fixação intramedular de fraturas, Fraturas do quadril

## Abstract

**Objective:**

Evaluate the stability provided by two flexible intramedullary nails (FINs) in a simulation of fractures at the proximal levels in pediatric femur models.

**Methods:**

Two FINs were inserted in 18 synthetic models of pediatric femurs. Fractures were simulated at one of three levels, and the models were divided into the following groups (n=6): diaphysis (control), subtrochanteric and trochanteric. Flex-compression tests were performed with force up to 85 N. Relative stiffness and the average deformation was obtained. Torsion tests were performed by rotating the proximal fragment until 20°, to obtain the average torque.

**Results:**

At flex-compression, the set’s average relative stiffness and average deformations were: 54.360x10^3^ N/m and 1.645 mm in the control group, respectively. In the subtrochanteric group, the relative stiffness was 31.415x10^3^ N/m (-42.2%) and the deformation was 2.424 mm (+47.3%) (p<0.05). For the trochanteric group, the relative stiffness was 30.912x10^3^ N/m (+43.1%) and the deformation was 2.508 mm (+52.4%) (p<0.05). In torsion, the average torque was 1.410 Nm in the control group; 1.116 Nm in the subtrochanteric group (-20.8%), and 2.194 Nm in the trochanteric group (+55.6%) (p<0.05).

**Conclusion:**

FINs do not seem to be biomechanically competent for the treatment of proximal femoral fractures. Level of Evidence I; Therapeutic Studies - Investigating the results of treatment.

## RESUMO

**Objetivo:**

avaliar a estabilidade proporcionada por duas hastes intramedulares flexíveis na simulação de fraturas nos níveis proximais em modelos pediátricos de fêmur.

**Métodos:**

Duas hastes foram inseridas em 18 modelos sintéticos de fêmures pediátricos. As fraturas foram simuladas em um dos três níveis, e os modelos foram divididos nos seguintes grupos (n=6): diáfise(controle), subtrocantérico e trocantérico. Testes de flexão-compressão foram realizados com força de até 85N. A rigidez relativa e a deformação média foram obtidas. Os testes de torção foram realizados girando o fragmento proximal até 20°, para obter o torque médio.

**Resultados:**

Na flexo-compressão, a rigidez relativa média e as deformações médias do conjunto foram: 54,360x10^3^ N/m e 1,645 mm no grupo controle, respectivamente. No grupo subtrocantérico a rigidez relativa foi de 31,415x10^3^ N/m (-42,2%) e a deformação foi de 2,424 mm (+47,3%) (p<0,05). Para o grupo trocantérico a rigidez relativa foi de 30,912x10^3^ N/m (+43,1%) e a deformação foi de 2,508 mm (+52,4%) (p<0,05). Na torção, o torque médio foi de 1.410 Nm no grupo controle; 1,116 Nm no grupo subtrocantérico (-20,8%) e 2,194 Nm no grupo trocantérico (+55,6%) (p<0,05).

**Conclusão:**

As hastes intramedulares flexíveis não parecem ser biomecanicamente competentes para o tratamento das fraturas proximais do fêmur. Nível de Evidência I; Estudos terapêuticos - Investigação dos resultados do tratamento.

## INTRODUCTION

Femur fractures represent approximately 1.6% of all bony lesions in children, with a 2.6 to 3.0 times higher incidence in boys than in girls.^1 , 2^ In the last few decades, treatment strategies for these fractures have moved from a conservative management to a more rational approach that favours surgical treatment as a result of the development of implants specially designed for children. Therefore, conservative treatment is now reserved for infants, and surgical fixation is predominantly used for older children and adolescents.^3^

The stabilization of diaphyseal fractures of the femur with a flexible intramedullary nail (FIN) inserted percutaneously decreases morbidity, provides stability, and does not disrupt callus formation.^3^ The method is effective, quick, and easyly performed, with minimal complications to treat diaphyseal fractures of the immature femur.^4^ Therefore, currently, FIN is used for treating patients older than 4 years of age or, even younger, when comorbidities are present such as obesity, spasticity, or poor bone quality.^5^

However, the utility of FINs is restricted when the fracture is excessively comminuted or when it is not located in the diaphysis of the bone.^6 , 7^ Some studies tried to establish the limits for using FINs when the fracture is located in the distal metaphysis or in the subtrochanteric region. Volpon et al. created a bone defect at the transition site between the diaphysis and distal metaphysis in models of pediatric femur and found that the stability provided by FINs was not significantly compromised.^8^ However, when the fracture is situated at proximal metaphysis the use of FINs is controversial.^9 , 10 , 11^

Based on this premise, the objective of the present investigation was to evaluate the mechanical stability provided by two FINs in simulated fractures (osteotomies) at the subtrochanteric and trochanteric regions in pediatric femur models.

## METHODS

Eighteen femur models of synthetic bone corresponding to the femur of a nine-year-old child (Sawbone Inc., Pacific Research Laboratories Inc., WA, United States) were used. This synthetic bone has been validated for its mechanical properties being similar to those of human bones.^12 , 13^

Flexible titanium nails (Titanium Elastic Nail - TEN®, TiGa 114v, DePuy Synthes®, Switzerland) were used to fix osteotomies created at three levels in the femur models. The nails had a diameter of 3.5 mm, corresponding to approximately 74% of the diameter of the medullary canal. The implants were inserted in a retrograde manner and each model was then radiographed to ensure the correct positioning of the nails.

After nail insertion, the models randomly received a cut (osteotomy) at one of three levels to simulate the location of the fractures. Three groups were created (n=6 each): trochanteric (osteotomy at the level of the lesser trochanter); subtrochanteric (osteotomy 3.5 cm distal to the lesser trochanter); and the control (osteotomy at the mid-diaphysis).^14^ Each osteotomy was complete and had an oblique inclination of 20° in the craniocaudal direction in the sagittal plane.

**Figure 1:**
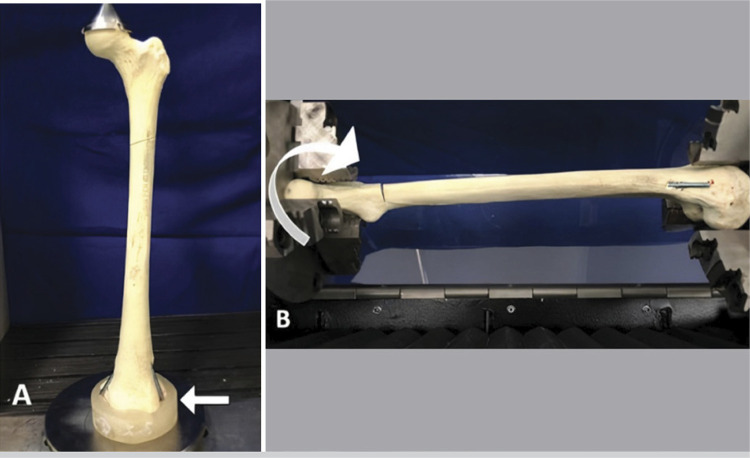
Mechanical tests. A - For flex-compression, the distal end of the femur was fitted into an acrylic model (white arrow), and a metal cup to simulate the acetabulum was fitted on the femoral head to receive the progressive vertical force. B - Torsion set up. The distal femur was held fixed and the proximal third was rotated externally until 20°.

The groups were subjected to flex-compression in a universal testing machine (DL 10000; load cell 50 kgf, EMIC, PR, Brazil). The bone models were placed in an upright position but with a slight inclination of 7^o^ in the varus position to simulate the *in vivo* load.^15^ An axial compression force of up to 85.0 N was applied to the femoral head at a speed of 0.1 mm/s ( Figure 1 A ). The relative stiffness and deformation were obtained.

The same sets were placed into a torsion machine (55 MT; Instron®, MA, USA), so that the distal end was fixed and the proximal end rotated externally at a rate of 0.5°/s until 20^o^ of rotation was attained ( Figure 1 B ) with determination of the torque.

### Statistical analysis

Measurements of the central tendency of the mechanical tests, variability, and position were estimated. A normality test was performed by the Shapiro-Wilk method. The deformation (mm) and torque (Nm) variables were approached using analysis of variance and Tukey’s test. The relative stiffness variable (N/m) was analysed by a Kruskal-Wallis test for multiple comparisons and by a Dunn test to determine the groups with significant differences. A significance level of 5% (p<0.05) was established. The software used was R, version 3.6.3 (The CORE TEAM, 2020, Vienna, Austria).

## RESULTS

**Figure 2:**
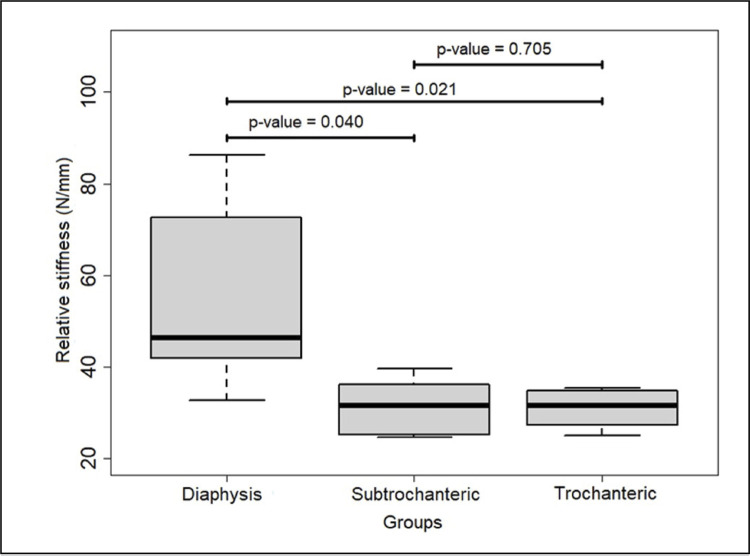
Box plot of relative stiffness in flexo-compression test. p-value<0.05 indicates statistical significance. Figure is presented as median and interquartile range.

**Figure 3:**
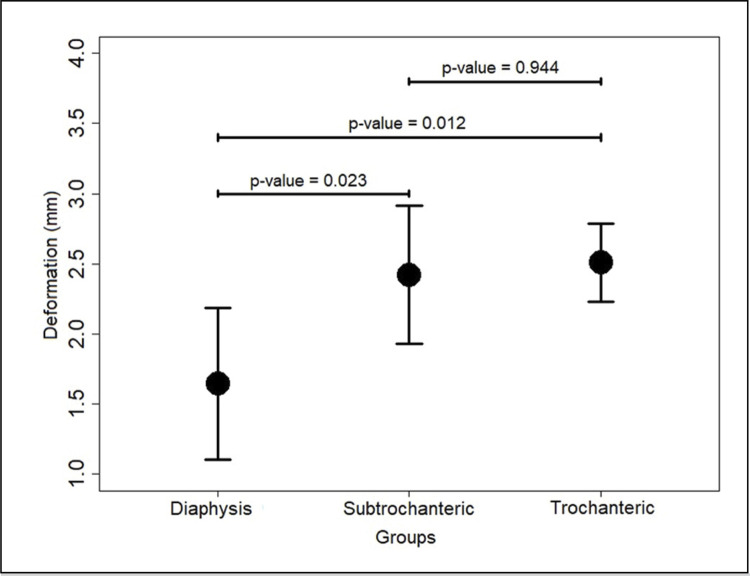
Error plot of deformation in flexo-compression test; p-value<0.05 indicates statistical significance. Figure is presented as mean and standard deviation.

In flex-compression, the relative stiffness and deformation are shown in Table 1 . At 85N, the relative stiffness values in trochanteric and subtrochanteric groups showed no difference between them (p>0.05), but their values were different from that of the control group (p<0.05) ( Figure 2 ). The deformation in trochanteric and subtrochanteric groups was not different (p>0.05), but they were different from the control group (p<0.05) ( Figure 3 ).

**Table 1:** Overall results of the mechanical tests.

Groups	Mean	Median	SD	Minimum	Maximum	IQR
	**Relative stiffness (x 10^3^ N/m)**					
Diaphysis	54.360	46.335	20.569	36.610	86.230	24.005
Subtrochanteric	31.415 (-42.2%)	31.510	6.416	24.590	39.590	10.198
Trochanteric	30.912 (-43.1%)	31.505	4.076	25.090	35.350	5.938
	**Deformation (mm)**					
Diaphysis	1.645	1.743	0.540	0.923	2.364	0.676
Subtrochanteric	2.424 (+47.3%)	2.358	0.492	1.805	3.016	0.750
Trochanteric	2.508 (+52.4%)	2.447	0.277	2.198	2.956	0.310
	**Torque (Nm) - 20^o^ angulation**					
Diaphysis	1.410	1.478	0.322	0.906	1.749	0.410
Subtrochanteric	1.116 (-20.8%)	1.037	0.281	0.825	1.601	0.279
Trochanteric	2.194 (+55.6%)	2.178	0.123	2.030	2.390	0.104

SD indicates standard deviation IQR, interquartile range.

**Figure 4:**
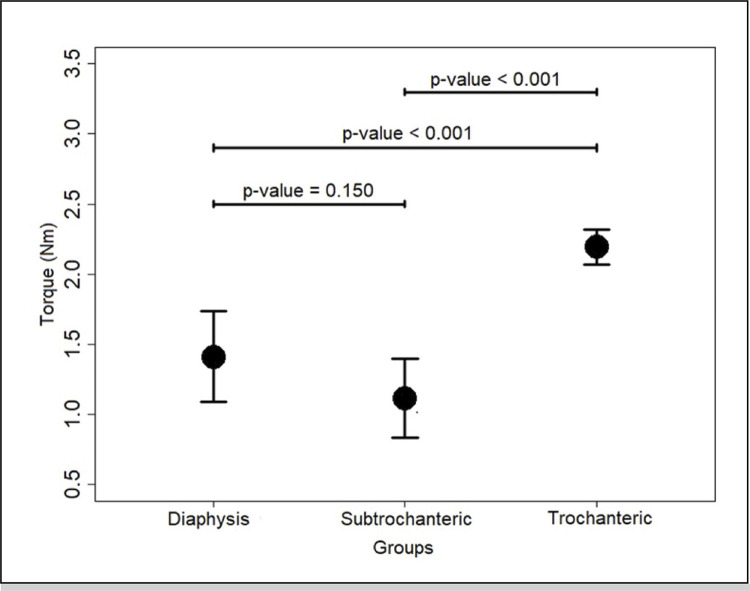
Error plot of torque in torsion test; p-value<0.05 indicates statistical significance. Figure is presented as mean and standard deviation.

The mean torque values at 20° of rotation are shown in table 1 . The control and subtrochanteric group values were significantly different from that of the trochanteric group (p<0.05), but there was no difference between the former two (p>0.05) ( Figure 4 ).

## DISCUSSION

Clinically, the fixation of fractures at the diaphysis of the femur with flexible intramedulary nails give good and uneventful outcomes, but the complications increase as the fracture is located more proximally.^10 , 11^ Therefore, we simulated fractures at that level fixed with two flexible nail implants.

In fractures at the subtrochanteric region the proximal fragment tends to flex, abduct, and externally rotate due to the muscular action. There is also a strong mechanical lever caused by the angle between the diaphysis and femoral neck, which causes a substantial tendency to produce a varus deformity.^16^ Such specific conditions lead to extraordinary mechanical demand on the implant. Clinical data reported by Li et al. on the effect of major complications of elastic nails on the stabilization of the subtrochanteric fractures^9^ support our results of flex-compression tests, which showed that a deformation in that region was around +149.8% of the value obtained in the mid-diaphyseal region. But we could not ascertain the ultimate limit of the deformation and the failure point of the systems because we worked in the mechanical elastic phase only. Other authors confirmed these findings and concluded that plates are better implants for proximal femoral fractures than flexible nails.^10 , 11^

However, Xu et al. compared the fixation of subtrochanteric fractures in children with FIN and locking plates and found that two methods can result in good functional outcomes.^11^ Some auhors presented a series of cases treated with FINs and showed that fracture located in the proximal region of the femur presented good outcome in children and adolescents and recommended using this technique.^17 , 18^ Nevertheless, the number of patients were relatively limited in both reports, and the authors used spica casting or knee bracing to provide an extra stabilization.^17 , 18^ More recently, Basa *et al.* evaluated 20 patients with subtrochanteric fracture treated with FIN and showed that all fractures obtained excellent or satisfactory results.^19^ Furthermore, reduction loss was a less common complication than originally thought in paediatric patients.^19^

In the flexion-compression test a load of 85.0 N was applied to the systems. This value was selected after taking into account the mass of the non-weight bearing lower limb (approx. 8.5 kg). It was previously used by Volpon et al.;^8^ additionally, this load allowed the deformation to be restricted to the elastic phase of the nails, thus fulfilling the objective of the study.

In torsion we found that the group of femurs with osteotomy in the most proximal region presented greater stiffness at 20° of rotation (+ 155% of the value in the control group). This may be explained by the greater contact area between the proximal and distal fragments in the area of cancellous bone. Neverheless, this parameter should be evaluated together with the other findings and not in isolation.

Our study has some limitations. First, both FINs were inserted retrograde. Maybe an anterograde insertion of one nail or penetrating the apophysis of the greater trocanter could increase the stability.^6 , 20^ Second, we tested the implant behavior based on the forces applied without accounting for partial weight-bearing, and, third, mechanical tests do not mimic real fractures because the soft tissue influence are not considered. However, when mechanical tests are used, it has been found that they provide a good correlation with clinical results. In addition, they provide information that spare time and contribute to patient’s safety.

## CONCLUSION

Our results suggest that the stability provided by FINs in groups of proximal osteotomies may not be adequate for the use of these implants. Therefore, recommendation of using two FINs for treating children’s proximal femur fractures is not supported by our results, and when using 3.5 mm flexible nails to stabilize subtrochanteric fractures, the addition of a brace or cast for 4 weeks is prudent.
